# A New Mixed-Backbone Oligonucleotide against Glucosylceramide Synthase Sensitizes Multidrug-Resistant Tumors to Apoptosis

**DOI:** 10.1371/journal.pone.0006938

**Published:** 2009-09-09

**Authors:** Gauri A. Patwardhan, Qian-Jin Zhang, Dongmei Yin, Vineet Gupta, Jianxiong Bao, Can E. Senkal, Besim Ogretmen, Myles C. Cabot, Girish V. Shah, Paul W. Sylvester, S. Michal Jazwinski, Yong-Yu Liu

**Affiliations:** 1 Department of Basic Pharmaceutical Sciences, University of Louisiana at Monroe, Monroe, Louisiana, United States of America; 2 Department of Cellular Biology and Anatomy, Louisiana State University Health Sciences Center, Shreveport, Louisiana, United States of America; 3 Department of Pathology, Louisiana State University Health Sciences Center, Shreveport, Louisiana, United States of America; 4 Department of Biochemistry and Molecular Biology, Medical University of South Carolina, Charleston, South Carolina, United States of America; 5 Experimental Therapeutics, John Wayne Cancer Institute, Santa Monica, California, United States of America; 6 Department of Medicine, Tulane University School of Medicine, New Orleans, Louisiana, United States of America; 7 Department of Biochemistry, Nantong University School of Medicine, Nantong, Jiangsu, China; National Cancer Institute, United States of America

## Abstract

Enhanced ceramide glycosylation catalyzed by glucosylceramide synthase (GCS) limits therapeutic efficiencies of antineoplastic agents including doxorubicin in drug-resistant cancer cells. Aimed to determine the role of GCS in tumor response to chemotherapy, a new mixed-backbone oligonucleotide (MBO-asGCS) with higher stability and efficiency has been generated to silence human GCS gene. MBO-asGCS was taken up efficiently in both drug-sensitive and drug-resistant cells, but it selectively suppressed GCS overexpression, and sensitized drug-resistant cells. MBO-asGCS increased doxorubicin sensitivity by 83-fold in human NCI/ADR-RES, and 43-fold in murine EMT6/AR1 breast cancer cells, respectively. In tumor-bearing mice, MBO-asGCS treatment dramatically inhibited the growth of multidrug-resistant NCI/ADR-RE tumors, decreasing tumor volume to 37%, as compared with scrambled control. Furthermore, MBO-asGCS sensitized multidrug-resistant tumors to chemotherapy, increasing doxorubicin efficiency greater than 2-fold. The sensitization effects of MBO-asGCS relied on the decreases of gene expression and enzyme activity of GCS, and on the increases of C_18_-ceramide and of caspase-executed apoptosis. MBO-asGCS was accumulation in tumor xenografts was greater in other tissues, excepting liver and kidneys; but MBO-asGCS did not exert significant toxic effects on liver and kidneys. This study, for the first time *in vivo*, has demonstrated that GCS is a promising therapeutic target for cancer drug resistance, and MBO-asGCS has the potential to be developed as an antineoplastic agent.

## Introduction

Chemotherapy remains a standard treatment for patients with metastatic cancers. However, multidrug resistance (MDR) often occurs in more than 50% of patients with cancers during the course of chemotherapy, ultimately resulting in treatment failures [1], [2]. Overexpression of genes that modulate drug action, cell proliferation and apoptosis is the cornerstone for MDR. Recent studies indicated that glucosylceramide synthase (GCS) is a gene for drug resistance in cancer cells [3]–[6]. GCS enzyme converts ceramide to glucosylceramide, thereby deactivating ceramide [7]. Ceramide, a lipid second messenger, mediates growth arrest and apoptosis of cells; ceramide-induced apoptosis contributes to the therapeutic efficiencies of anthracyclines, taxanes, *Vinca* alkaloids, cytokines, and irradiation [4], [8]–[10]. Transfection of GCS gene confers cellular resistance to doxorubicin, tumor necrosis factor-α and daunorubicin in various cancer cell lines [11]–[13]. GCS overexpression has been identified in MDR cell lines of breast, ovarian, cervical, and colorectal cancers [14], [15]. GCS has been found overexpressed in leukemia patients with poor-response to chemotherapy [16], [17] and GCS overexpression is associated with the prognosis of breast cancer [18]. Furthermore, a number of studies over past decade have demonstrated that inhibition of GCS sensitizes MDR cells to anticancer drugs [3], [15], [19]–[26]. Suppressing GCS overexpression using small interfering RNA (siRNA), phosphorothioate antisense oligonucleotide (PS-oligo) and transfection of antisense sequence overcomes MDR in human breast, colon, cervical and ovarian cancer cell lines [3], [15], [20], [23], [24], [26]. Inhibition of GCS enzyme with small molecules, such as D-*threo*-1-phenyl-2-decanoylamino-3-morpholino-1-propanol (PDMP), sensitizes cancer cells to doxorubicin, paclitaxel and vincristine [19], [21], [22], [25].

Emerging evidence indicates that ceramide glycosylation is a newly identified mechanism promoting cellular resistance; however, whether GCS directly modulates tumor response to chemotherapy remains to be clarified. Effectively silencing the GCS gene *in vivo* can determine whether GCS has a role in tumor drug resistance. Mixed-backbone oligonucleotide (MBO), one type of second-generation antisense oligonucleotides, can specifically inhibit the expression of pathogenic genes and has a better safety profile than PS-oligo *in vivo* [27], [28]. Using the strategy of gene silencing with MBO, we examined the role of GCS in cancer drug resistance.

## Materials and Methods

### Reagents and cell lines

A mixed-backbone oligonucleotide (MBO) was designed to target the open reading frame (ORF) 18–37 of human GCS [23], [29] and designated as MBO-asGCS. A scrambled control (MBO-SC) had the same chemical components as MBO-asGCS, but no sequence specificity. MBOs were 20-mer phosphorothioate DNA, excepting four bases at either the 5′ end or the 3′ end was replaced by 2′-O-methyl RNA. MBOs and Cy3-labeled MBO-asGCS were synthesized, and purified by reverse-phase HPLC and desalting (Integrated DNA Technologies, Inc., Coralville, IA). Lipofectamine^TM^ 2000, Opti-MEM I, and NBD C_6_-ceramide (*N*-hexanoyl-D-erythro-sphingosine) complexed to BSA were purchased from Invitrogen (Carlsbad, CA). Doxorubicin hydrochloride was purchased from Sigma. Anti-human GCS rabbit serum (GCS 6.2) [30] was kindly provided by Drs. D. L. Marks and R. E. Pagano (Mayo Clinic and Foundation, Rochester, MN). Anti-GCS goat IgG was purchased from Santa Cruz Biotechnology (Santa Cruz, CA) and anti-active caspase-7 rabbit IgG was from Calbiochem (La Jolla, CA). C_6_-Ceramide (*N*-hexanoyl-D-erythro-sphingosine) and NBD C_6_-glucosylceramide were purchased from Matreya (Pleasant Gap, PA).

Human breast adenocarcinoma cell line MCF-7 and drug-resistant NCI/ADR-RE (previously designed as MCF-7-AdrR) [31], [32] were kindly provided by Dr. Kenneth Cowan (UNMC Eppley Cancer Center, Omaha, NE) and Dr. Merrill Goldsmith (National Cancer Institute, Bethesda, MD). Murine breast carcinoma cell line EMT6 and its drug-resistant counterpart EMT6/AR1 [33], [34] were kindly provided by Dr. Ian Tannock (Ontario Cancer Institute, Toronto, ON, Canada). MCF-7 and NCI/ADR-RE cells were maintained in RPMI-1640 medium, and EMT6 and EMT6/AR1 cells were maintained in Dulbecco's modified eagle medium (DMEM). Both media were supplied with 10% fetus bovine serum (FBS), 100 units/ml penicillin, 100 µg/ml streptomycin, and 584 mg/liter L-glutamine. Cells were cultured in an incubator humidified with 95% air and 5% CO_2_ at 37°C. EMT6/AR1 cells were cultured in medium containing 1 µg/ml of doxorubicin for 2 days/week in addition to the above components.

### MBO uptake

Cy3-labeled MBO-asGCS was used to analyze MBO uptake, as described previously with modification [35]. Briefly, cells (5×10^4^ cells/well) were seeded in 24-well plates and cultured in 10% FBS RPMI-1640 medium. After 24 hr growth, cells were exposed to 50 nM Cy3-MBO-asGCS with Lipofectamine^TM^ 2000 in Opti-MEM I reduced-serum medium for defined periods. After washing with ice-cold PBS three times and addition of methanol (200 µl/well), cellular fluorescence was measured at λ_excitation_ 550 nm/λ_emission_ 570 nm using a Synergy HT multi-detection microplate reader (BioTek, Winnooski, VT). MBO-asGCS uptake was normalized by cell numbers and represented by the percentage of cellular fluorescence of total fluorescence added in the medium before incubation. To characterize the *in vivo* uptake of MBO-asGCS, Cy3-MBO-asGCS was administrated by intraperitoneal injection (1∼4 mg/kg) into tumor-bearing mice. Tissues were collected 7 hr after injection, and the fluorescence in tissue homogenates was measured in the same manner as described for cells.

### Cell viability assay

Cell viability was analyzed by quantitation of ATP, an indicator of active cells, using the CellTiter-Glo luminescent cell viability assay (Promega, Madison, WI), as described previously [15]. Briefly, cells (4,000 cells/well) were grown in 96-well plates with 10% FBS RPMI-1640 medium for 24 hr. MBOs were introduced into cells by Lipofectamine 2000 (vehicle control) in Opti-MEM I reduced-serum medium, for a 4 hr incubation. Cells were then incubated with increasing concentrations of agents in 5% FBS medium for additional 72 hr. Cell viability was determined by the measurement of luminescent ATP in a Synergy HT microplate reader, following incubation with CellTiter-Glo reagent.

### Tumor xenografts and treatments

All animals were handled in strict accordance with good animal practice as defined by AAALAC, and all animal work was approved by the IACUC, University of Louisiana at Monroe (ULM). A drug-resistant tumor model was established with the protocols described previously [36], [37]. Athymic nude mice (*Foxn1^nu^/Foxn1^+^*, 4–5 weeks, female) were purchased from Harlan (Indianapolis, IN) and maintained in the ULM vivarium. Cells of NCI/ADR-RE (3–5 passages) were washed with and resuspended in serum-free RPMI-1640 medium. Cell suspensions (1×10^6^ cells in 20 µl per mouse) were injected into the second left mammary gland, just beneath the nipple. Mice were monitored by measuring tumor growth, body weight and clinical observation. Once tumors reached ∼2 mm in diameter, mice were randomly divided into treatment and control groups (ten mice per group). MBO-asGCS or MBO-SC, dissolved in RPMI 1640 medium was injected at a dose of 1 mg/kg, twice per week, at the tumor site. The control group received medium only (saline). Doxorubicin was administered by intraperitoneal injection, at 2 mg/kg once a week. In combinations, doxorubicin was administered with medium (saline) or MBOs, respectively.

### RNA extraction and GCS mRNA analysis

Cellular RNA was extracted and purified using a SV total RNA isolation kit (Promega). Equal amounts of RNA (100 ng) were used for RT-PCR and a 441-bp GCS fragment was produced using a SuperScript^TM^ One-step RT-PCR with Platinum *Taq* kit (Invitrogen), as described previously [13], [15]. The levels of GCS mRNA were semi-quantitated by optical densitometry and normalized using the OD values of glyceraldehyde-3-phosphate dehydrogenase (GAPDH). For quantitative RT-PCR, cDNA was synthesized using the SuperScript^TM^ First-Strand synthesis system and random hexamer reverse transcription primers (Invitrogen). Under upstream primer (5′-GACCTGGCCTTGGAGGGAAT-3′) and downstream primer (5′-GAGACACCTGGGAGCTTGCT-3′) conditions, a 149-bp fragment in the region of GCS gene (303 to 451) was produced using a QuantiFast SYBR Green PCR kit (Qiagen, Valencia, CA) with a MyiQ real-time PCR detection system (Bio-RAD Laboratories, Hercules, CA), as described previously [15]. Endogenous GAPDH (200 bp; upstream primer 5′-ATGGGGAAGGTGAAGGTCGG-3′; downstream primer 5′-TCCACCACCCTGTTGCTGTA-3′) was used for normalization. Quantitation was carried out using human GCS DNA standard curves generated by a serial dilution of pcDNA 3.1-GCS plasmid [3], [20].

### Western blot analysis

After treatments, cells or tissue homogenates were lysed using NP40 cell lysis buffer (Biosource, Camarillo, CA). Equal amount of proteins (50 µg/lane) were resolved using 4–20% gradient SDS-PAGE (Invitrogen). The transferred blots were blocked with 5% fat-free milk PBS and immuno-blotted with primary antibodies (anti-GCS goat IgG or anti-active caspase-7 rabbit IgG) at 1∶500 dilution, at 4°C for overnight, as described previously [3], [13], [15]. The antigen-antibody in blots was detected by using a second antibody-conjugated horseradish peroxidase (HRP) and enzyme-linked chemiluminescence (ECL) plus substrate (GE Healthcare, Piscataway, NJ). Endogenous GAPDH was used as a loading control. The levels of GCS protein were represented by the ratios of optical densities in GCS bands normalized against GAPDH.

### Immunohistochemistry

Tumors were removed, fixed and maintained in paraffin blocks. Microsections of tumors (5 µm) were stained in H&E and identified by pathologists (Dr. Bao, J., Pathology, Louisiana State University Health Sciences Center). For immunostaining, antigens were retrieved in steaming sodium citrate buffer (10 mM, 0.05% Tween-20, pH 6.0; for 10 min). After blocking in 2% block solution (Vector Laboratories, Burlingame, CA), the slides were incubated with anti-GCS rabbit serum (1∶100) overnight at 4°C. Antibody-bound cells on slides were recognized by Alexa Fluor^®^488 goat anti-rabbit IgG (Invitrogen). Cell nuclei were counterstained with 4′, 6-diamidino-2-phenylindole (DAPI) in mounting solution (Vector Laboratories). The slides were observed using a Nikon TE-2000 phase contrast microscope, and the images were captured by a Retiga 2300^TM^ monochrome digital camera using IPLab^TM^ image analysis program (Scanalytics Inc., Rockville, MD).

### Cellular ceramide glycosylation assay

Cells (1×10^6^ cells/dish) were grown 24 hr in 35-mm dishes with 10% FBS RPMI-1640 medium, and MBO-asGCS (50 nM) was then introduced as described above. After 12 hr of growth in 10% RPMI-1640 medium, cells were switched to 1% bovine serum albumin (fatty acid free) medium containing 500 µM NBD C_6_-ceramide complexed to BSA (Invitrogen). After a 2 hr incubation at 37°C, lipids were extracted, and resolved by partisil high-performance TLC plates with fluorescent indicator with solvent of chloroform/methanol/3.5 N ammonium hydroxide (85∶15∶1), as described previously [15]. NBD C_6_-glucosylceramide and NBD C_6_-ceramide were identified using an AlphaImager HP imaging system (Alpha Innotech, San Leandro, CA), and quantitated with a Synergy HT multi-detection microplate reader (BioTek). For quantitation, calibration curves were established after TLC separation of NBD C_6_-ceramide and NBD C_6_-glucosylceramide.

### High performance LC/MS ceramide measurement

The levels of endogenous ceramides in tumors were measured using normal phase high performance liquid chromatography coupled to atmospheric pressure chemical ionization-mass spectrometry (LC/MS) as described previously [38], [39]. After MBO treatment (1 mg/kg, twice per week for 38 days), doxorubicin was given by intraperitoneal injection at 2 mg/kg, and tumor tissues collected at the indicated periods after doxorubicin administration. The ceramide levels were normalized against phosphorus in tissues.

### Caspase-3/7 assay

Caspase-3/7 activity was assayed by DEVD-aminoluciferin cleavage, using the caspase-Glo^®^ 3/7 assay kit (Promega), following the manufacturer's instruction. Briefly, NCI/ADR-RE cells were cultured in 100-mm dishes (5×10^5^ cells per dish) with 10% FBS RPMI medium. After 24 hr of growth, MBO-asGCS was introduced into cells with Lipofectamine 2000 in Opti-MEM I reduced-serum medium. Cells were incubated for a 48 hr in 5% FBS medium containing 5 µM doxorubicin. After harvest, cell lysates were incubated with proluminescent DEVD-aminoluciferin and thermostable luciferase. The luminescence for each sample was measured using a Synergy HP multiplate reader and normalized by proteins. For *in-vivo* studies, tissue homogenates (25 mg/100 µl) from each group were immediately used for caspase-3/7 assay.

### Apoptosis analysis by flow cytometry

The analyses were performed using propidium iodide (PI) staining with subsequent FACS analysis, as described previously [40] with minimal modification. Cells (5×10^5^ per dish) were cultured in 100-mm dishes with 10% FBS RPMI 1640 medium for 24 hr. MBO-asGCS was then introduced into cells with Lipofectamine 2000 in Opti-MEM I reduced-serum medium. Cells were incubated in 5% FBS medium in the presence of 5 µM doxorubicin for additional 48 hr. After harvest with trypsinization and centrifugation, cell pellets were resuspended and exposed to 0.01% PI in staining solution (0.1% sodium citrate, 0.3% Triton X-100, 2 mg/ml ribonuclease A) at 4°C for 30 min, followed by flow cytometry analysis using FACSCalibur (BD Biosciences, San Jose, CA). Sub-phase G1/G0 was defined as indicative of apoptotic cells; 10,000 events were counted.

### Apoptotic cell death detection using terminal-deoxynucleotide-transferase-mediated dUTP nick end labeling (TUNEL) staining

Apoptotic cells were detected by measurement of nuclear DNA fragmentation using the DeadEnd fluorometric TUNEL system (Promega), following the manufacturer's instruction, as described previously [13], [23]. Briefly, cells (2×10^4^ per chamber) were cultured in 4-chamber slides with 10% FBS RPMI 1640 medium. MBOs were introduced into cells with Lipofectamine 2000 in Opti-MEM I reduced-serum medium (4 hr incubation). Cells were then incubated in 5% FBS medium in the presence of 5 µM doxorubicin for additional 48 hr. Cells were fixed with methanol digested for 20 min with 0.2 mg/ml proteinase K in 10 mM Tris-HCl, pH 8.0, and labeled for 90 min with fluorescein-12-dUTP terminal deoxynucleotide transferase reaction mixture at 37°C in a humidified chamber. After mounting with DAPI, slides were observed using a Nikon TE-2000 phase contrast microscope with digital image capture.

All experiments in cells were performed in triplicate and repeated at least two times. Data were analyzed by using Microsoft Excel 2003 and Prism (V.4) and presented as mean±SD. Tumor volume (V) was calculated by V = L×W^2^/2, where L was the length and W was the width of tumors. Statistically significant differences between samples were analyzed using two-tailed Student's *t* tests for paired and unpaired samples, p<0.05 was considered significant.

## Results

### MBO-asGCS suppresses GCS overexpression and sensitizes drug-resistant cancer cells

**Figure 1:**
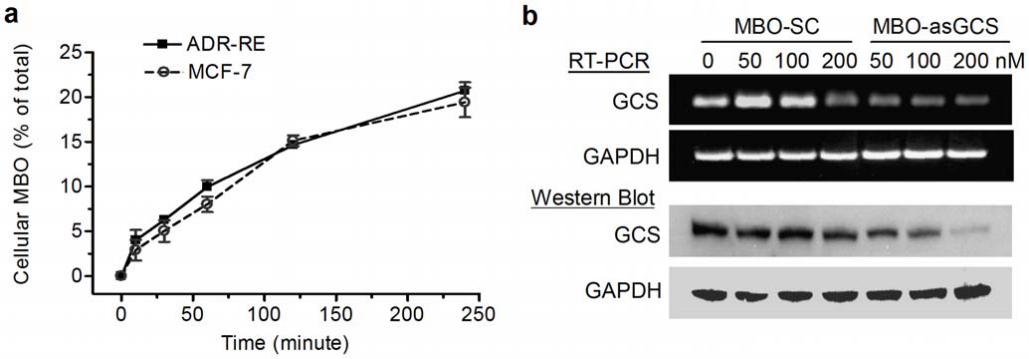
Mixed-backbone oligonucleotide targeting human glucosylceramide synthase. a. Cellular uptake of MBO. After exposure of cancer cells to Cy3-labeled MBO-asGCS (50 nM) for the indicated periods, cellular Cy3-MBO-asGCS was measured at λ_excitation_ 550 nm and λ_emission_ 570 nm. b. Influence of MBOs on GCS expression. MBO-asGCS or MBO-SC was introduced into NCI/ADR-RE cells (ADR-RE) with Lipofectamine 2000 in Opti-EME I reduced-serum medium. After 48 hr growth, total RNA and protein were extracted. Total RNA (100 ng/reaction) was analyzed by RT-PCR. For Western blots, total protein (50 µg/lane) was subjected to 4–20% SDS-PAGE electrophoresis. Proteins were transferred to nitrocellulose and immunoblotted with GCS primary antibody (1∶500) and detected using ECL plus. GCS protein levels were presented as the ratios of the optical densities in GCS bands normalized against GAPDH.

MBO-asGCS has been designed to target the exon-1 of human GCS gene [23], [29]. The influence of MBO-asGCS and MBO-SC (scramble control) have been examined in NCI/ADR-RE cells, which overexpress GCS and display MDR [3], [31], [32]. We found that resistant NCI/ADR-RE cells took up approximately the same amount, 20% of total Cy3-MBO-asGCS, as drug-sensitive MCF-7 cells (Fig. 1a) in 4 hr of transfection. Similar uptake for MBO-asGCS also has been found in drug-resistant EMT6/AR1 and drug-sensitive EMT6 murine breast cancer cell lines (21.8% *vs.* 20.3% in 4 hr). As shown in Fig. 1b, MBO-asGCS, not MBO-SC, decreased GCS gene expression as measured by mRNA and protein levels, in a dose-dependent manner. At low concentration (50 nM), MBO-asGCS decreased the GCS expression to 40% of the levels resulting with MBO-SC treatment.

**Figure 2:**
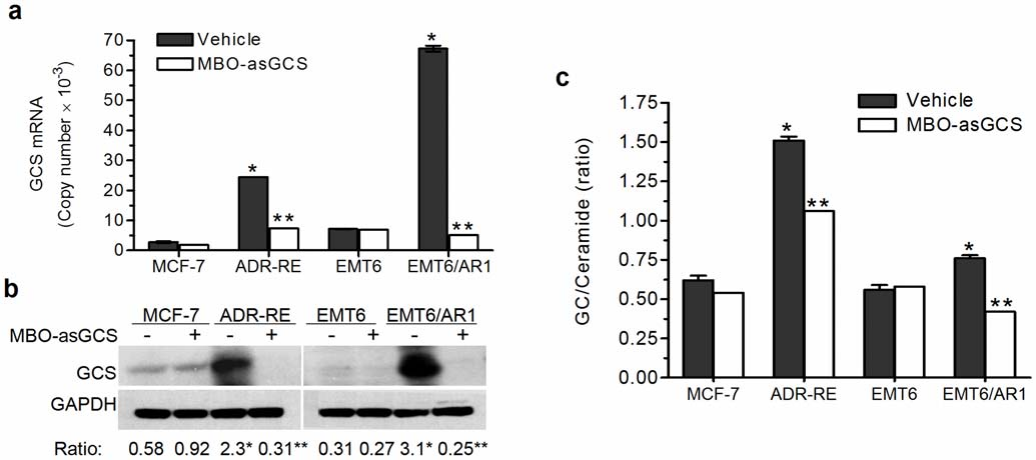
MBO-asGCS suppresses GCS expression in drug-resistant cancer cells. Drug-resistant and drug-sensitive cell lines (NCI/ADR-RE, MCF-7; EMT6/AR1, EMT6) were treated with MBO-asGCS (50 nM) for 48 hr. a. Quantitative RT-PCR. Isolated total RNA (100 ng/reaction) was synthesized to cDNA and analyzed by quantitative real-time PCR. b. Western blot. Total protein (50 µg/lane) was subjected to Western blot analysis; GCS expression levels are presented as the density ratio of GCS/GAPDH bands. c. Cellular ceramide glycosylation. After 24 hr MBO transfection, cells were incubated 500 µM NBD C_6_-ceramide complexed to BSA. After 2 hr incubation, cellular sphingolipids were extracted and resolved by high-performance thin-layer chromatography and quantitated. ADR-RE, NCI/ADR-RE; *, p<0.001 compared with drug-sensitive cells; **, p<0.001 compared with corresponding vehicle control.

We examined the effects of MBO-asGCS on GCS in drug-resistant and drug-sensitive cancer cell lines. MDR murine EMT6/AR1 breast cancer cells [34], like NCI/ADR-RE, overexpressed GCS mRNA and protein (Fig. 2a, 2b). Consistent with increases of mRNA levels and enzyme activities (Fig. 2c), GCS protein levels in NCI/ADR-RE and EMT6/AR1 were 4-fold (2.3 *vs.* 0.58 ratio of GCS/GAPDH) and 10-fold (3.1 *vs.* 0.31) greater than in sensitive MCF-7 and EMT6 cells, respectively. MBO-asGCS treatment (50 nM) significantly decreased ceramide glycosylation following a substantial decrease of GCS expression in resistant NCI/ADR-RE and EMT6/AR1 cells. The levels of GCS protein as well as mRNA were decreased to approximately 13% in NCI/ADR-RE (0.31 *vs.* 2.3) and 8% in EMT6/AR1 cells (0.25 *vs.* 3.1); cellular GCS activities (as assessed by GC/Cer ratios) were reduced to approximately 65% and 54% in NCI/ADR-RE and EMT6/AR1, respectively (Fig. 2c). However, MBO-asGCS did not significantly affect GCS expression in drug-sensitive MCF-7 and EMT6 cells (Fig. 2). Given that drug-resistant and drug-sensitive cells can take up the same amount of MBO-asGCS (Fig. 1a), these data indicate that MBO-asGCS selectively suppress GCS overexpression in drug-resistant cells.

**Figure 3:**
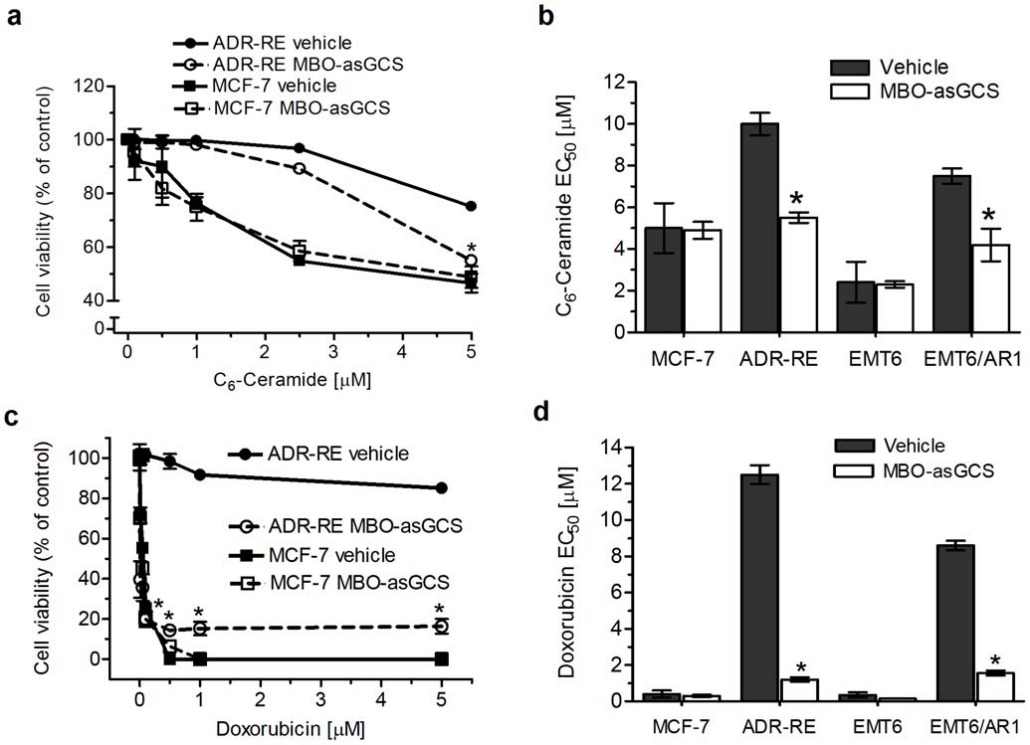
MBO-asGCS increases doxorubicin sensitivity in drug-resistant cancer cells. Cells (4,000/well) were plated in 96-well plates and pretreated with MBO-asGCS (50 nM). After 24 hr growth, cells were shifted to 5% FBS medium containing increasing concentrations of agents and grown for additional 72 hr. Cell viability was measured using the CellTiter-Glo luminescent cell viability assay. a. Cell viability after C_6_-ceramide treatment. ADR-RE, NCI/ADR-RE cells; *, p<0.01 compared with vehicle treatment. b. EC_50_ values for C_6_-ceramide. *, p<0.001 compared with vehicle treatments. c. Cell viability after doxorubicin treatment. d. EC_50_ values for doxorubicin. *, p<0.001 compared with vehicle treatment.

Additional studies showed that MBO-asGCS significantly increased cytotoxicity of ceramide and doxorubicin in drug-resistant cells, but not in drug-sensitive cells. MBO-asGCS pretreatment (50 nM) did not increase ceramide cytotoxicity in drug-sensitive MCF-7 and EMT6 cells. In contrast, MBO-asGCS (50 nM) significantly increased ceramide cytotoxicity in drug-resistant NCI/ADR-RE and EMT6/AR1; the EC_50_ values for C_6_-ceramide decreased to approximately 50% in both resistant cell lines (Fig. 3a, 3b). MBO-asGCS pretreatment markedly increased doxorubicin sensitivity in drug-resistant cells; the EC_50_ values for doxorubicin decreased by 83-fold (0.18 *vs.* 12.5 µM) in NCI/ADR-RE, and by 43-fold (0.20 *vs.* 8.6 µM) in EMT6/AR1, respectively (Fig. 3c and 3d). By comparison, MBO-asGCS only mildly (by 50%) decreased the EC_50_ values for doxorubicin in sensitive counterparts of MCF-7 and EMT6 cells. These results demonstrate that suppressing GCS overexpression sensitizes resistant cancer cells to therapeutic agents, such as doxorubicin whose therapeutic efficiency is associated with ceramide actuation [11], [41], [42].

### MBO-asGCS promotes MDR cells to induced-apoptosis

**Figure 4:**
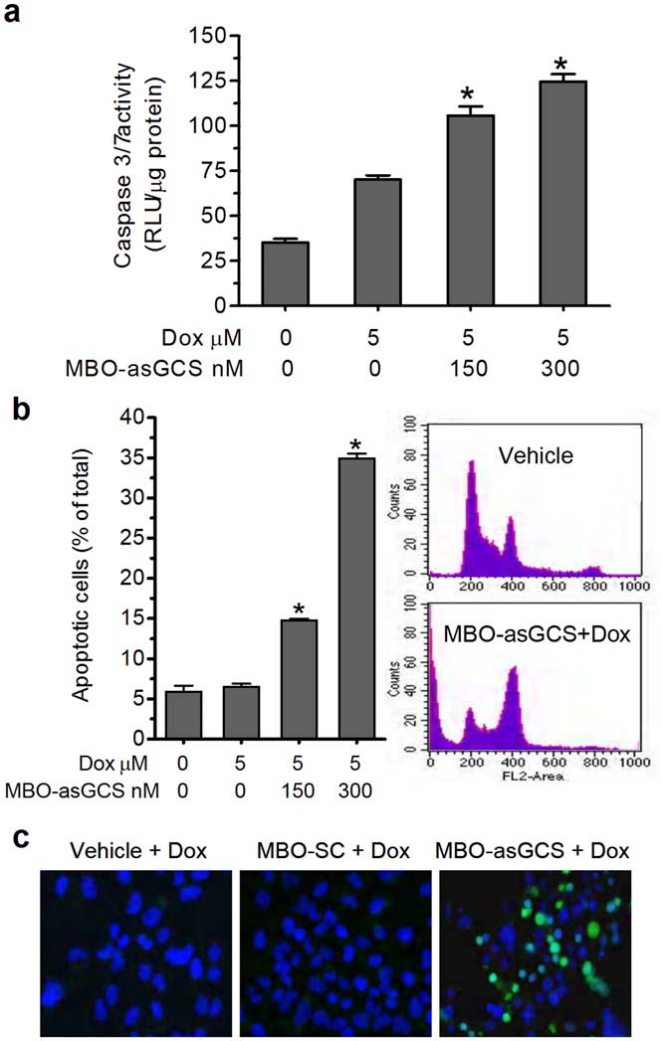
MBO-asGCS enhances doxorubicin-induced apoptosis. NCI/ADR-RE cells were pretreated with MBO-asGCS (0–300 nM) and then exposed to doxorubicin (Dox. 5 µM) for 48 hr. a. Caspase-3/7 assay. RLU, relative luminescence units; *, p<0.005 compared with cells exposed to doxorubicin alone. b. Flow cytometry analysis. Apoptosis was quantitated by flow cytometry following propidium iodide staining (right panel). Left-hand bar graph is based on apoptotic cells detected on the sub-G0 phase. *, p<0.001 compared with cells exposed to doxorubicin alone. c. TUNEL staining for apoptosis. After pretreatment of MBO-asGCS or MBO-SC (50 nM), NCI/ADR-RE cells were exposed to doxorubicin (5 µM) for 48 hr. Apoptotic cells (TUNEL^+^) exhibit green fluorescence (x 200).

The apoptotic impacts of anthracyclines and taxanes depend, at least in part, on ceramide generation [3], [43]–[45]. We assessed the effects of MBO-asGCS treatment on ceramide-induced apoptosis in MDR cells exposed to doxorubicin. It was found that doxorubicin exposure induced apoptosis only in MDR cells pretreated with MBO-asGCS. Doxorubicin increased caspase 3/7 activity, in a dose-dependent manner, in NCI/ADR-RE cells pretreated with MBO-asGCS (Fig. 4a). Correspondingly, flow cytometry detected large proportions of apoptotic cells in drug-resistant NCI/ADR-RE cells pretreated with MBO-asGCS and then with doxorubicin, but not in cells treated with doxorubicin alone (Fig. 4b). MBO-asGCS pretreatment increased the number of apoptotic cells to 225% (14.7 *vs.* 6.5% of total of cells) and 533% (34.9 *vs.* 6.5% of total cells), at 150 nM and 300 nM, respectively, as compared with doxorubicin treatment alone. Furthermore, in TUNEL assays, combined pretreatment of MBO-asGCS following with doxorubicin increased the apoptotic fraction by 6-fold (30% *vs.* 5% of total cells), as compared with treatments of doxorubicin alone or doxorubicin following MBO-SC pretreatment (Fig. 4C). Given that MBO-asGCS suppressed ceramide glycosylation of GCS (Fig. 2) increasing cellular ceramide, these data indicate that MBO-asGCS promotes MDR cancer cells to apoptosis through ceramide-activated caspases.

### MBO-asGCS sensitizes MDR tumors to doxorubicin

**Figure 5:**
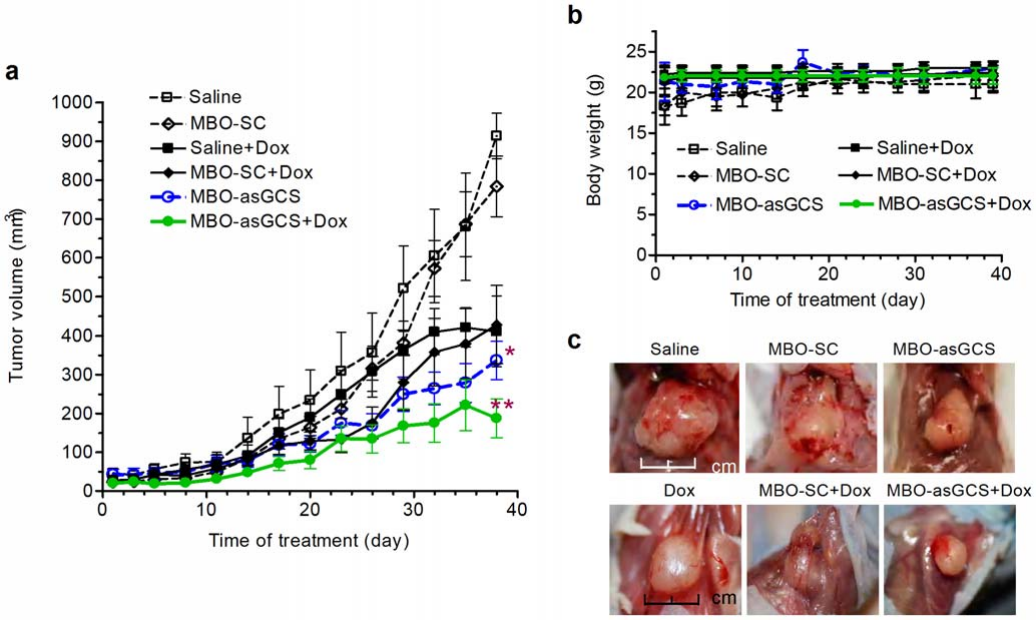
MBO-asGCS suppresses tumor growth in athymic nude mice. Athymic nude mice (*Foxn1^nu^/Foxn1^+^*, 10 per group) bearing drug-resistant tumors (NCI/ADR-RE cells) were treated with either MBOs (1 mg/kg every 3 days, intratumoral injection) alone or MBOs combined with doxorubicin (Dox, 2 mg/kg once a week, intraperitoneal injection) for 38 days. Treatments were started once tumors were visible (2 mm diameter, day 0). a. Tumor growth. *, p<0.001 compared with treatments in the presence of saline or MBO-SC. **, p<0.001 compared with treatments in the presence of doxorubicin or doxorubicin combined with MBO-SC. b. Body weight of mice after treatments. c. Tumors after treatments. Tumors were photographed when mice were sacrificed at the end of treatment regimens.

**Figure 6:**
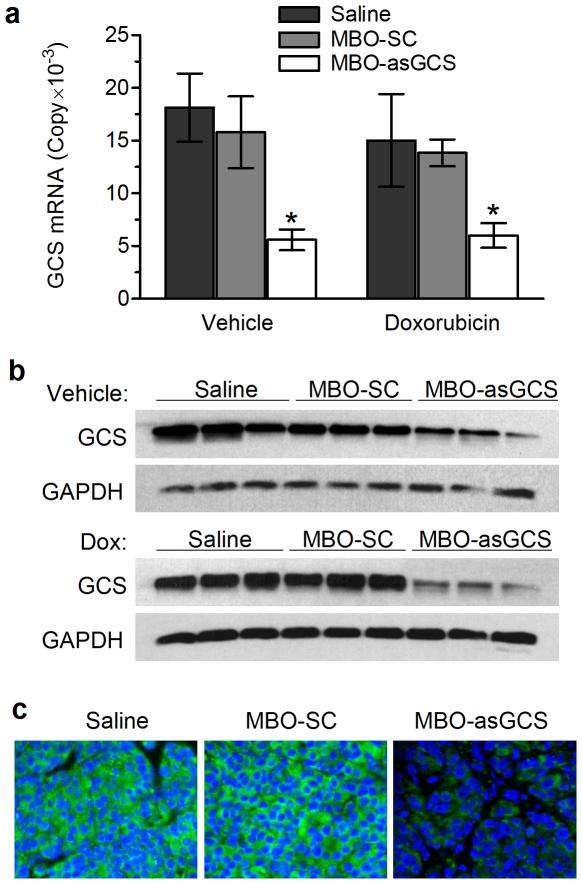
MBO-asGCS suppresses GCS expression in tumors. Mice bearing NCI/ADR-RE tumors were treated with MBOs alone (1 mg/kg per 3 days, intratumoral injection) or in combination with doxorubicin (Dox, 2 mg/kg per week, intraperitoneal injection) for 38 days. Total RNA and proteins were immediately extracted from tumor homogenates. a. Quantitative RT-PCR for GCS. GCS mRNA levels were quantitated by using GCS standard and normalized against GAPDH. *, p>0.001 compared with saline or MBO-SC groups. b. Western blotting for GCS. Detergent-soluble protein (50 µg/lane) extracted from tumors (three per group) was incubated with anti-GCS or anti-GAPDH antibodies, following PAGE and transferring. c. Immunofluorescence microscopy. Tumor sections were stained with anti-GCS antibody (green). The nuclei were visualized by staining with DAPI (blue, x 200).

In order to validate whether MDR tumors rely on ceramide glycosylation for evading toxicity, we assessed the effects of MBO-asGCS on tumor growth and tumor response to chemotherapy in nude mice. Treatment was started when MDR tumors became visible (∼2 mm in diameter), approximately two weeks after inoculation of NCI/ADR-RE cells (10^6^ cells/mouse). After 13 administrations of MBO (1 mg/kg, intratumoral injection, every three days, 10 mice/group), it was found that MBO-asGCS, but not MBO-SC treatment, significantly attenuated tumor growth to 37% (336±49 *vs.* 913±58 mm^3^, p<0.01) (Fig. 5a). On contrary, MBO-SC could not significantly affect tumor growth (783±78. *vs.* 914±58 mm^3^) (Fig. 5a). Furthermore, it was found that MBO-asGCS treatment sensitized MDR tumors to doxorubicin. Combined treatment of MBO-asGCS with doxorubicin decreased tumor volume to 45% (187±50 *vs.* 411±90 mm^3^, p<0.01), as compared with the treatment of doxorubicin or doxorubicin combined with MBO-SC (411±90 mm^3^; 428±100 mm^3^). Moreover, these treatments did not significantly affect the body weight of these mice. After 39 days of treatment, the mean of body weight was 22.0±1.0 g in the group of MBO-asGCS combination with doxorubicin, as compared to 21.0±1.0 in the saline group (Fig. 5b). It was also observed that the combined treatment of MBO-asGCS with doxorubicin distinctly isolated tumor from around tissues, in contrast to the other treatment groups (Fig. 5c). Three lung metastases were found in the saline as well as in the MBO-SC control groups (10 mice/group), but none in the MBO-asGCS treatment groups. Further assessments showed that MBO-asGCS specifically suppressed GCS expression in tumors (Fig. 6). MBO-asGCS decreased GCS mRNA to approximately 30% (5,591 *vs.* 18,130 copies; 6,006 *vs.* 15,005 copies) in tumors, as compared with saline or treatment with doxorubicin alone (Fig. 6a); however, MBO-SC treatment did not significantly modulate GCS mRNA levels in either the presence or absence of doxorubicin (Fig. 6a). The effects of MBO-asGCS on GCS expression were further confirmed by GCS protein changes detected using Western blotting and immunostaining (Fig. 6b, 6c).

**Figure 7:**
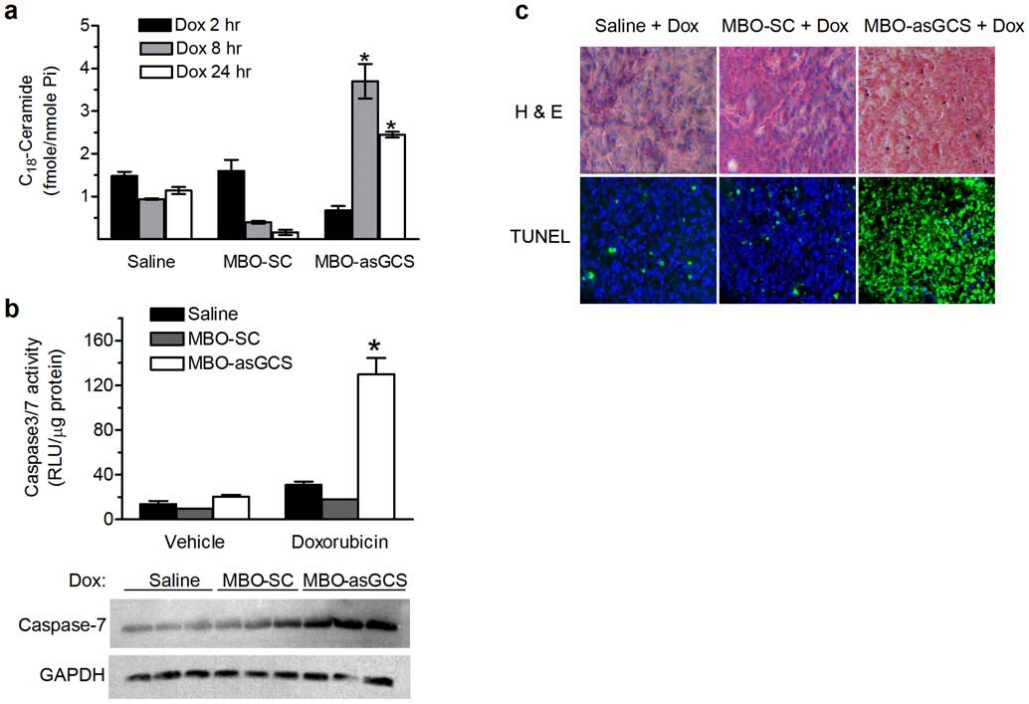
MBO-asGCS leads to ceramide-induced apoptosis *in vivo*. Mice bearing NCI/ADR-RE tumors were treated with MBOs (1 mg/kg, intratumoral), doxorubicin (Dox, 2 mg/kg, ip), or combinations for 3 days. a. Ceramide analysis. Lipids were extracted from tumors treated with MBOs (1 mg/kg every 3 days, 3 times) and indicated periods of doxorubicin treatments (Dox). Endogenous C_18_-ceramide was normalized to phosphorus (Pi). *, p<0.001 compared with combination of MBO-SC and doxorubicin. b. Caspase-3/7 assay. After combination treatments of MBOs and Dox, tumors were resected 48 hr after Dox injection. *Upper bar graph*, Caspase-3/7 activities in tumors were measured by using caspase-3/7 assay as described in Methods. RLU, relative luminescence units; *, p<0.001 compared with doxorubicin treatment. *Lower panel*, active form of caspase-7 was detected by Western blot. c. Apoptosis. Successive sections of tumors after treatments were stained with H&E, or subjected to TUNEL assay. Apoptotic cells (TUNEL^+^) exhibit green fluorescence (x 200).

We characterized dynamic changes of tumor ceramides via an LC/MS assay. We found that doxorubicin exposures for 24 hr enhanced C_18_-ceramide accumulation more than 4-fold (2.4 *vs.* 0.67 fmole/nmole Pi) in MDR tumors treated with MBO-asGCS; however, doxorubicin alone or combined with MBO-SC could not significantly affect C_18_-ceramide levels in tumors (Fig. 7a). Correspondingly, the combination of MBO-asGCS and doxorubicin increased caspase 3/7 levels by 4-fold greater (130 *vs.* 31 RLU/µg protein) and significantly enhanced amounts of active form of caspase-7 detected by Western blotting (Fig. 7b). This MBO-asGCS combined treatment also substantially increased the number of apoptotic cells in MDR tumors, as detected by a TUNEL assay (Fig. 7c). By contrast, doxorubicin alone or a combination of doxorubicin with MBO-SC did not significantly increase caspase 3/7 or apoptosis in MDR tumors.

**Figure 8:**
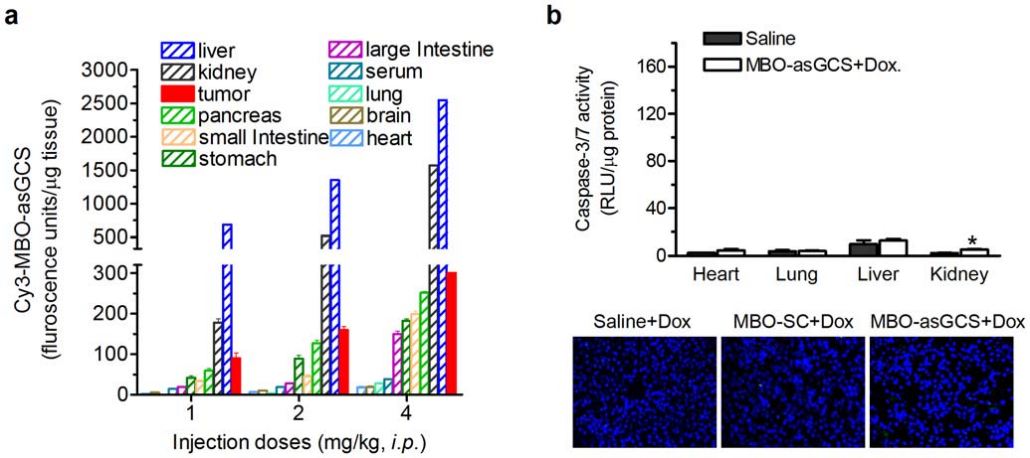
Accumulations and effects of MBO-asGCS in normal tissues. a. MBO-asGCS accumulations. After 7 hr injection of Cy3-labeled MBO-asGCS (ip, 3 mice/each), tissues were removed and homogenized immediately. Fluorescence was measured and normalized against tissue weight. b. Apoptosis analysis. Mice were treated with combination of MBOs (1 mg/kg every three days, ip, for three times) and Dox (2 mg/kg, ip, for 48 hr; 3 mice/group). *Upper bar graph*, Caspase-3/7 activities in heart, lung, liver and kidney were assessed by using caspase-3/7 assay. RLU, relative luminescence units; *, p<0.05 compared with saline control. *Lower panel*, apoptotic cells in kidney were detected by using TUNEL staining.

We assessed the accumulations of Cy3-labeled MBO-asGCS after intraperitoneal administration (1 mg/kg, sampling 7 hr post-dose). As shown in Fig. 8a, we found that MDR tumors took up approximately 0.8% of MBO-asGCS and that amount was greater than these in other tissues (pancreas, small intestine, stomach, large intestine, serum, lung, brain, heart), excepting liver and kidneys that are the major organs for oligonucleotide degradation (5.8% in liver, 0.99% in kidney). After 48 hr of treatments, we examined caspase-executed apoptosis in tissues. There were no significant changes in caspase activity or apoptotic cells in lungs, heart and liver of mice treated with MBO-asGCS combined with doxorubicin (Fig. 8b). There was a 2-fold increase in caspase-3/7 activity (p>0.05) and a 3% increase in apoptotic cells in the kidneys of mice treated with MBO-asGCS and doxorubicin, as compared with the saline control group; however, these increases were no significant differences, as compared with doxorubicin treatment groups (Fig. 8b).

## Discussion

In the present study, we examine the effect of MBO against GCS on cancer drug resistance. These results demonstrate, for the first time, that suppressing GCS overexpression specifically reverses drug resistance, and attenuates tumor progression.

Overexpressed GCS has been found in drug-resistant cancer cells and in tumors [3], [14]–[17], [46], [47]; however, whether ceramide glycosylation by GCS constitutes a significant mechanism by which tumors develop the resistance has been less studied. GCS are overexpressed in MDR cancer cell lines of human breast (MCF-7-P500), cervix (KB-A1), ovary (A2780-AD), colon (SW620AD) and leukemia (K56/A02, HL-60/ADR) that have been selected by anthracycline [14]–[17], [48]. GCS overexpression was also found in MDR murine EMT6/AR1 breast cancer cells in this study (Fig. 2). In addition to GCS, overexpression of several other genes including *MDR1* and *Bcl-2*, and mutant tumor suppressor p53 are known to cause these cells resistance, particular sample as NCI/ADR-RE cells [3], [14], [31]. Efficient inhibition of GCS in NCI/ADR-RE cells *in vivo* thus offered the opportunity to prove and clarify GCS roles in cancer drug resistance. We employed MBO as a specific tool in this study, since this second-generation antisense oligonucleotide displays higher efficiency and more stability than PS-oligo *in vivo* [27], [49]. We found that MBO-asGCS enhances doxorubicin sensitivity by 83-fold and 43-fold in MDR NCI/ADR-RE and EMT6/AR1 cells, respectively, and by 220% in MDR tumor xenografts of NCI/ADR-RE. Given that MBO-asGCS accumulates more in tumors (Fig. 8a) and equally in both drug-resistant and -sensitive cells (Fig. 1a), it is reasonably concluded that drug resistance of tumors, at least of some, depend on GCS overexpression. Targeting GCS can eliminate tumors with poor response to conventional chemotherapy, such as doxorubicin. The suppression of GCS by MBO-asGCS restores ceramide signaling (particular C_18_-ceramide) during the course of doxorubicin treatment, thereby promoting caspase-executed apoptotic death in cells and in tumors. These data demonstrate that ceramide glycosylation by GCS plays a key role in MDR tumor survival and growth, thus GCS is an important target for improving cancer chemotherapy.

Previous studies have shown that several approaches inhibiting GCS reverse drug resistance in cancer cells, however, a potential therapeutic agent that efficiently inhibits GCS *in vivo* remains to be developed. One of GCS inhibitors, PDMP overcomes drug resistance in cell lines of breast, ovarian and colon cancer [19], [21], [22], [25]. However, like others, the specificity and efficiency of PDMP is limited, as the active site and the catalytic mechanism of GCS have not yet been well characterized [50]–[53]. Transfection of siRNA or antisense sequence (full length) has been found to specifically silence the GCS gene and sensitize MDR cells to several first-line anticancer drugs [3], [20], [26]. Inadequacy of delivery and low levels of therapeutic gene expressed *in vivo*, which are encountered with any gene therapy, limit the use of siRNA and antisense gene transfection *in vivo* studies. Our previous work showed that PS-oligo specifically suppresses GCS expression and efficiently reversed drug resistance in cells [15], [23]. A MBO that is modified by addition of several 2′-*O*-methylribonucleotides in DNA sequence has significantly improve the *in-vivo* stability, binding affinity, and biodistribution of oligos [27], [54], [55]. Indeed, we found that the new MBO-asGCS targeting human GCS reported herein, efficiently suppressed GCS expression and sensitized MDR tumor to doxorubicin in mice. More interestingly, tracking Cy3-labeled MBO-asGCS in mice revealed that MDR tumors took up 0.8% of administered MBO-asGCS; This tumor uptake level is greater than in other normal tissues (pancreas, small intestine, stomach, large intestine, serum, lung, brain, heart), excepting liver and kidneys. Assessments of caspase-executed apoptosis suggest that MBO-asGCS can significantly augment doxorubicin cytotoxicity in MDR tumors (Fig. 7), but not in kidney (Fig. 8). These results suggest that MBO-asGCS is an efficient agent to suppress GCS overexpression specifically in MDR tumors.

In conclusion, the present work primarily demonstrates that GCS overexpressed in cancers, at least in metastatic breast cancer, represents a viable and likely important target for the treatment of drug-resistant cancers. MBO-asGCS constitutes a specific and effective GCS inhibitor and appears to have great potential to be developed to an antineoplastic agent.
